# General practitioner practices in requesting laboratory tests for patients with gastroenteritis in the Netherlands, 2001–2002

**DOI:** 10.1186/1471-2296-7-56

**Published:** 2006-10-02

**Authors:** Winette E van den Brandhof, Aad IM Bartelds, Marion PG Koopmans, Yvonne THP van Duynhoven

**Affiliations:** 1 Department of Infectious Diseases Epidemiology, National Institute of Public Health and the Environment (RIVM), Bilthoven, The Netherlands; 2 Netherlands Institute for Health Services Researches (NIVEL), Utrecht, The Netherlands; 3 Department of virology, National Institute of Public Health and the Environment (RIVM), Bilthoven, The Netherlands

## Abstract

**Background:**

The objective of this study was to estimate the (selective) proportion of patients consulting their GP for an episode of gastroenteritis for whom laboratory tests were requested. In addition adherence of GPs to the guidelines for diagnostic test regime was ascertained.

**Methods:**

Data were collected from a GP network in the Netherlands. Information was also collected on the reason for requesting the test, test specifications, and test results.

**Results:**

For 12% of the GP patients with gastroenteritis, a stool sample was requested and tested for enteric pathogens. In most patients, the duration, followed by severity of complaints or a visit to a specific, high-risk country were reported as reasons to request laboratory diagnostics. Tests were requested most often in summer months and in February. Campylobacter (requested for 87% of the tests), Salmonella (84%), Shigella (78%) and Yersinia (56%) were most frequently included in the stool tests. Campylobacter was detected most often in patients.

**Conclusion:**

Test requests did not always comply with existing knowledge of the etiology of gastroenteritis in GP patients and were not always consistent with the Dutch GP guidelines. Therefore, the data of this study can be used to develop educational approaches for GP's as well as for revision of the guidelines.

## Background

Approximately 4.5 million episodes of gastroenteritis occur every year in the Netherlands [1]. The incidence of consultations in general practices (GP) is estimated at 14 per 1,000 person years, yielding an annual estimate of approximately 220,000 consultations for gastroenteritis [2]. For an unknown number of these patients, diagnostic laboratory tests will be requested. Patients with a positive diagnostic test result within the service area of the Laboratory Surveillance Infectious diseases network are reported in a system for *Campylobacter *spp, and *Salmonella *spp [3]. For *Shigella *spp and *Escherichia coli *O157 notification is mandatory. A separate laboratory-based sentinel system exists for a selection of viral pathogens [4].

In 1996, guidelines for patients with acute diarrhoea were created by the Dutch college of GPs to improve daily practice [5]. Guidelines try to limit laboratory test to patients that benefit from knowing the etiology for therapeutic purposes and try to limit tests to the pathogens that are more likely to be present. There is a general restrictive policy in the Netherlands with regard to requesting tests, using medicines and referrals to specialists. Besides good practice these guidelines help to (among others) reduce costs and burden for health care and patients.

Furthermore, a general-practice based study was performed in the Netherlands from 1996 to 1999, to investigate the etiology of gastroenteritis [6]. The results of this study did not completely support the guidelines, and it is unknown whether the Dutch GPs were influenced by the results of this study.

Within the broad framework of large-scale studies on the incidence and etiology of gastroenteritis in the community and GPs in the Netherlands, the current study was performed to estimate the proportion of patients consulting their GP for an episode of gastroenteritis for which specified laboratory tests were requested. With these data, annual estimates can be made of the number of GP consultations for gastroenteritis as a whole by using the laboratory surveillance data (especially number of stool samples submitted), assuming no change in diagnostic testing practices. In addition, we collected information on the reason for requesting the test and on test results. Information on the factors that influence GPs diagnostic practices for patients with gastroenteritis (i.e. which patients are selected to submit stool samples?) are needed to more accurately interpret laboratory-based surveillance data.

These data can also be used in the assessment of adherence to the current national guidelines for GP practices for management of acute gastroenteritis. These guidelines were developed before the results of large-scale studies on gastroenteritis were known, and thereby possibly need revision.

## Methods

The study was performed in the Netherlands in 2001 and 2002, in co-operation with the sentinel general practice (GP) network of the Netherlands Institute for Health Services Research (NIVEL). The network consists of on average 45 practices that cover 1% of the Dutch population, representative with regard to age, gender, regional distribution, and degree of urbanisation. The general practitioners are representative for other GPs in the Netherlands, and are motivated to participate in monitoring the work of the GPs in this country.

Since 1996, all practices have reported the number of consultations for gastroenteritis by age group, gender, practice, and week of consultation [6]. In 2001 and 2002, they additionally reported when they ordered laboratory diagnostics for a gastroenteritis patient. Three weeks later, the administration of the NIVEL sent a questionnaire to the GP to obtain demographic data of the patient, the reason why the GP ordered laboratory diagnostics, for which pathogens diagnostics had been ordered, the result of the tests, and whether antibiotics had been prescribed.

The guidelines of the Dutch college of GPs for patients with acute diarrhoea state:

• Request laboratory diagnostics for patients with severe complaints and for patients who are at risk to spread the disease;

• With severe complaints, test for Salmonella, Campylobacter and Shigella;

• When a patient has symptoms for more than ten days, test for protozoa, especially for children, when visit to a foreign country is reported and in patients with decreased resistance;

• When the symptoms are acute, tests for Yersinia or viruses should not be performed.

Descriptive analyses were performed using SAS version 8. Cross tabulations were made for the laboratory requests and outcomes and demographic characteristics of the patients, week of consultation, and the reason for requesting laboratory diagnostics. Statistical differences were, where appropriate, tested by using the Chi-square test or Fisher exact test if the expected value in one of the cells was less than five.

## Results

**Figure 1:**
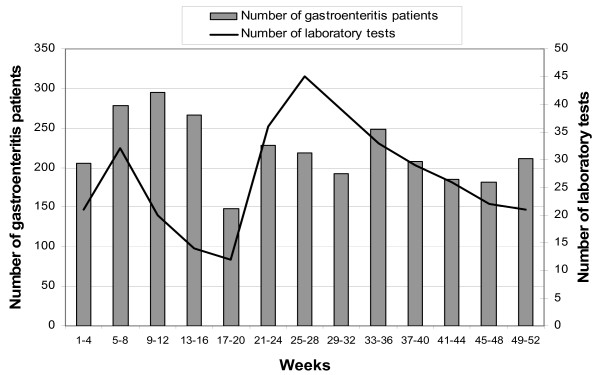
Number of gastroenteritis patients and number of laboratory tests per 4-week period, 2001 and 2002 combined.

In 2001, the GPs reported 1,464 patients with gastroenteritis. For 177 of these patients (12%), they ordered laboratory diagnostics. In 2002, 173 laboratory tests among 1,403 patients (12%) were ordered. For young children, relatively fewer laboratory tests were requested (8–10% of the patients), but in general, differences in percentages were rather small (table 1). There were no differences according to degree of urbanization or region (data not shown). Laboratory tests were more often requested in the summer months (weeks 21 to 32, test requested for 16–21% of patients with gastroenteritis). There was a smaller second peak in February (Figure 1).

**Table 1:** Number of gastroenteritis patients consulting a GP^a^, incidence per 10,000 persons, and number of laboratory diagnostics, by age, 2001 and 2002 combined.

	**Gastroenteritis patients consulting a GP**	**Incidence (per 10,000 persons)**	**Number of laboratory diagnostics (% of patients)**
**Total**	2,867	95.0	350 (12%)
			
**Age in years**			
0	197	663.7	19 (10%)
1–4	536	373.4	55 (10%)
5–14	434	124.8	33 (8%)
15–24	323	91.1	44 (14%)
25–39	559	72.6	79 (14%)
40–64	568	59.1	91 (16%)
≥65	250	60.7	29 (12%)

a) GP = general practitioner

For 258 (74%) of the total of 350 requests of laboratory diagnostics, questionnaires were returned. The GP ordered laboratory diagnostics for 15 patients who eventually did not supply a faecal sample to the laboratory (6%). These patients were not included in further analyses. The remaining 243 patients were representative with regard to degree of urbanisation, regional distribution and 4-week period of the total of 350 requests. For newborns, relatively few questionnaires were received (for 11 of 19 requests, 58%).

### Test reasons

For 90 patients (37%) more than one reason for the requested laboratory diagnostics was reported. Duration of complaints was reported most often (table 2). Also, severity of complaints, specific complaints (such as bloody diarrhoea) and a visit to a high-risk country were common reasons (table 2). Of the 53 times a visit abroad was reported as the reason, Turkey (n = 12, 23%) and Egypt (n = 10, 19%) were the most common travel destinations. For five patients, diagnostics were requested because of their profession (in the catering industry or childcare). For patients 65 years and older, duration and severity of complaints were reported significantly more often as reason for faecal testing (Chi-square test p = 0.01). For patients between 40 and 64 years, a visit abroad was significantly more often the reason (Chi-square test p < 0.05). For children aged 1 to 4 years more faecal tests were requested to reassure the parents compared to other age groups (Chi square test p = 0.02). For men a visit to a specific country was reported significantly more often as the reason for faecal testing (28% (n = 27) vs. 15% (n = 18) for women, Chi-square test p = 0.02). No further statistically significant differences were observed for the reasons of testing by comparing sexes.

**Table 2:** Reason of requesting laboratory diagnostics, by age, 2001 and 2002 combined

**Age in years**	**Total**	**0**	**1–4**	**5–14**	**15–24**	**25–39**	**40–64**	**65+**
								
**Reason**								
Duration of complaints	141 (58%)	6 (55%)	16 (46%)	11 (50%)	16 (48%)	40 (68%)	35 (56%)	17 (85%)
Severity of complaints	54 (22%)	2 (27%)	6 (17%)	5 (23%)	8 (24%)	11 (19%)	10 (16%)	11 (55%)
Visit to specific country	53 (22%)	2 (18%)	5 (14%)	4 (18%)	4 (12%)	13 (22%)	23 (37%)	2 (10%)
Reassurance (of parents)	42 (17%)	2 (18%)	11 (31%)	5 (23%)	7 (21%)	6 (10%)	8 (13%)	3 (15%)
Specific complaints	25 (10%)	0	2 (6%)	4 (18%)	5 (15%)	6 (10%)	7 (11%)	1 (5%)
Other reason	23 (9%)	0	3 (9%)	3 (14%)	3 (9%)	5 (8%)	7 (11%)	2 (10%)

### Pathogens included in test request

For many patients, the GP requested tests for Campylobacter as well as Salmonella, Shigella and Yersinia (49 times) (table 3). The second most common combination was diagnostics for these four pathogens together with *Giardia lamblia *(33 times). Tests for only Campylobacter, Salmonella and Shigella were ordered 30 times. Overall, tests for Campylobacter were requested most often (in 87% of the 243 requests for diagnostics), followed by tests for Salmonella (84%), Shigella (78%), Yersinia (56%) and *Giardia lamblia *(48%). In total, bacteria were included in 90% of the tests. Viruses were requested for 29 patients (12%): rotavirus 29 times (12%), in addition adenovirus 16 times (7%). Tests for parasites were requested for 125 patients (51%), most often *Giardia lamblia *(115 times) and Cryptosporidium (26 times).

**Table 3:** Pathogens requested for laboratory diagnostics, and number of positive tests, 2001 and 2002 combined

	**Number of tests**	**Positive (% of all tests/% of patients tested for the specific pathogen)**
**Total**	243	70 (29%)
		
**Pathogen**		
Campylobacter	212	33 (14%/16%)
Salmonella	205	9 (4%/5%)
Shigella	190	4 (2%/2%)
Yersinia	136	0
*Giardia lamblia*	115	13 (5%/11%)
Worms/Wormeggs/Cysts	35	3 (1%/9%)
Rotavirus	29	2 (0.8%/7%)
Cryptosporidium	26	1 (0.4%/4%)
Adenovirus	16	1 (0.4%/6%)
E. coli	7	1 (0.4%/14%)

### Tested pathogens in relation to age, sex and season

No substantial age, sex or seasonal differences were found for the requests of bacteria.

Newborns were significantly more often tested for viruses (45% (n = 5) vs. 10% (n = 24), Chi-square test p = 0.004). No differences by sex were observed in requesting viral tests. The proportion of viral tests among all tests was slightly higher between May and July (13% vs. 5% the rest of the year, Chi-square test p = 0.01). Finally, newborns also tended to be more often tested for Cryptosporidium (27%) than older patients (10%), although not statistically significant (Fisher exact test p = 0.11). No clear sex or seasonal differences were observed in requesting parasitic tests.

### Tested pathogens in relation to test reason

Yersinia was requested significantly often for patients tested because of the severity of complaints (Chi-square test p = 0.02). For the other bacteria the tendency was similar, but not significant (table 4). The GPs requested Shigella more often when reassurance was needed (Chi-square test p = 0.06). For other pathogens no association with the reason for testing was identified.

**Table 4:** Number of patients tested for a specific pathogen by reason of request, and number of positive tests, 2001 and 2002 combined. Percentages given are % of all tests for this reason, and % of these tests being positive.

**All (n = 243 patients)**	**Campylobacter**	**Salmonella**	**Shigella**	**Yersinia**	**Viruses**	**Parasites**
**Total/positive**	212/33	205/9	190/4	136/0	29/2	125/17
						
**Reason**						
Duration of complaints (n = 141)	124/20 (88%/16%)	121/8 (86%/7%)	113/4 (80%/4%)	81/0 (33%)	16/2 (11%/13%)	75/11 (53%/15%)
Severity of complaints (n = 54)	49/10 (91%/20%)	47/4 (87%/9%)	45/2 (83%/4%)	40/0 (74%)	7/1 (13%/14%)	25/4 (46%/16%)
Visit to specific country (n = 53)	46/5 (87%/11%)	46/1 (87%/2%)	43/3 (81%/7%)	33/0 (62%)	7/1 (13%/14%)	28/2 (53%/7%)
Reassurance (of parents) (n = 42)	39/5 (93%/13%)	39/0 (93%)	38/0 (90%)	25/0 (60%)	8/0 (19%)	19/0 (45%)
Specific complaints (n = 25)	21/10 (84%/48%)	15/1 (60%/7%)	14/1 (56%/7%)	9/0 (36%)	2/0 (8%)	8/2 (32%/25%)
Other reason (n = 23)	17/1 (74%/6%)	17/1 (74%/6%)	13/0 (57%)	9/0 (39%)	1/0 (4%)	14/4 (61%/29%)

### Test results and relation with test reason and season

For 64 patients (26%) test results were positive. With regard to test outcome, Campylobacter most often tested positive (n = 33, table 3). Thirteen patients had an infection with *Giardia lamblia*, nine a Salmonella-infection and four an infection with Shigella. Viruses were detected in only two patients (in one patient both rotavirus and adenovirus) (table 3). Patients who were tested because of the severity of their complaints more often were found to be positive for Campylobacter, rotavirus and Salmonella (table 4), although the differences were not statistically significant. Besides, patients who were tested because of the duration of their complaints more often tested positive for rotavirus, adenovirus, and Salmonella, although again no statistical significance was reached. Patients who had been in a high-risk country significantly more often were positive for Shigella (Fisher exact test p = 0.03). For adenovirus and rotavirus the tendency was the same, but not statistically significant. Patients who were tested because of specific complaints (especially bloody diarrhoea) significantly more often tested positive for Campylobacter (Fisher exact test p = 0.01).

For 179 patients (74%) test results were negative. For 91% (n = 10) of tested newborns no pathogens were found, while only 55% (n = 11) of tests for patients over 64 years were negative. In the winter (November-March), in 84% (n = 59) of the stools no pathogens were found. No differences were found for men and women. When a patient was tested because of reassurance, more often no pathogens were detected (n = 37, 88%).

The positivity rate of tests for bacteria was highest in the summer months (57% of all tests for bacteria yielded a positive result in weeks 21 to 36). For viruses, positive test results were too rare to study a relationship with season. For parasites no relationship between proportion of positive tests and season was observed.

### Prescription of antibiotics

For 64 patients (27%) antibiotics were prescribed for the symptoms of gastroenteritis. Only one of 11 newborns received antibiotics (9%), while antibiotics were prescribed to 40% of patients 65 years and older. For 21 patients (33%) no pathogens were found in the stool sample (table 5). It was not known however, whether test results were known when the antibiotics were prescribed. In total, for 62% (n = 29) of patients with a bacterial pathogen, for 50% (n = 1) of those with a viral pathogen and for 88% (n = 15) of those with a parasite, antibiotics were prescribed.

**Table 5:** Laboratory results of patients who had received antibiotics, 2001 and 2002 combined

	**Number of patients (% of patients who had received antibiotics)**
**Total**	64
	
**Results**	
No pathogens detected	21 (33%)
Bacteria^a^	29 (45%)
*Giardia lamblia*^a^	12 (19%)
Other parasites	3 (5%)
Viruses^a^	1 (2%)

a) In one patient *Giardia lamblia *and a bacterium were detected and in one other patient *Giardia lamblia *and a virus were detected

## Discussion

For 12% of the patients consulting their GP with an episode of gastroenteritis a stool sample was requested and tested for enteric pathogens. The duration, followed by severity of complaints or a visit to a specific, high-risk country were reported as the main reasons to request laboratory diagnostics. Tests were requested most often in summer months and in February. Campylobacter, Salmonella, Shigella and Yersinia were most frequently included in the stool tests, and Campylobacter was detected most often in patients (14%).

### Requesting stool tests

The IID-study team performed a similar study in England [7]. They found that 27% of the patients consulting their GP had a stool test performed. In that study, GPs were not asked why they requested the diagnostics, but clinical considerations were thought to play the most important role (and not surveillance purposes). In the Netherlands, a more reserved testing policy is pursued than in the United Kingdom, what could explain the lower percentage of patients with a stool sample request. In addition, gastroenteritis patients in the Netherlands visit their GP less often for an episode of gastroenteritis (5% vs. 17% in UK) [2,7]. Comparing the Netherlands to the United States and Canada, again the number of stool samples tested was relatively low: 9.1 per 1,000 inhabitants (average 1996–2001) [8], 16.8 per 1,000 [9] and 14.9 per 1,000 [10], respectively.

Fourty-four percent of the physicians in a survey in the United States had requested a stool culture for the last patient seen with acute diarrhoea [11]. This survey also included physicians specialized in internal medicine, emergency medicine and pediatricians, what might be one of the explanations for this higher percentage. Referral of a physician's patient by another provider increased the likelihood that a stool culture was requested. In this study, recent travel to a developing country, duration of diarrhoea more than 3 days, bloody stools and a diagnosis of AIDS were associated with a stool-culture request, quite similar to the factors observed in our country, although we had no information whether patients were diagnosed with AIDS. When, among others, physician speciality and reference from another provider was controlled for, travel to a developing country no longer was associated with a stool-culture request. It is unknown whether the survey included the peak of travel in the summer months. In another survey in the United States of patients in the general population, only 21% of patients reported that a stool culture had been requested [12]. The difference with the study of Hennessy *et al*. [11] is thought to result from recall bias and respondents in the general population may have thought that a stool culture request would be the desirable answer.

In the Netherlands, the national GP guidelines for patients with acute diarrhoea [5] state that uncomplicated cases can be dealt with by a phone consultation by a GP assistant. This GP assistant's advice lowers the number of patients visiting the GP and by that the number of stool requests. Besides, in the Netherlands, there is a relatively restricted antibiotic policy. Elucidation of the pathogen causing the gastroenteritis is therefore less important, since it does not always affect patient's treatment.

### Guidelines

The guidelines the GPs are provided with by the Dutch college of GPs [5] state that stool samples should be obtained of patients with severe gastroenteritis only (in connection with possible hospital admittance) and that they should be tested for Salmonella, Shigella and Campylobacter. In practice, for only 22% of the patients the severity of the complaints was given as the reason for testing. These patients were hardly tested more often for Campylobacter, Shigella and Salmonella. Test results in these types of stools were relatively more often positive for Salmonella, Shigella and Campylobacter, supporting these current guidelines.

Yersinia was rarely seen in patients with gastroenteritis [6], and is not recommended as a routine by the guidelines. Nevertheless, it was among the most common tested pathogens in this study.

The guidelines also state that faeces should be tested for protozoa in patients with duration of diarrhoea longer than ten days, especially children, after a stay in high-risk countries or in patients with decreased resistance. However, in practice protozoa were not requested more often for stools collected because of the duration of the complaints of the patients or because of a travel history. Requesting protozoa tests for more susceptible patients is only reflected in a relatively frequent request to test for Cryptosporidium in newborns.

Relatively few tests for Yersinia were requested for patients who were tested because of long duration of the complaints. Whether the remaining tests for Yersinia were only requested for patients with acute gastroenteritis is not known. This is also true for the testing of viruses.

Not following the guidelines does not always have to be the choice of the GP. In our study, in almost 90% of the tests requested to reassure a patient (or the parents) no pathogens were detected compared to 74% overall.

### Revision of the guidelines needed?

In the study performed from 1996 to 1999 [6], hardly any tests were positive for Shigella (0.1%). Therefore a test for Shigella should be less often requested (limited to patients with travel history), in contrast to the current guidelines.

The guidelines do not mention that samples from young children should also be tested for rotavirus (especially in the winter), although previous studies showed a high percentage of young patients testing positive for rotavirus [1,13]. Unbridled testing on bacteria can be prevented if the chance of a viral pathogen, considering the age and season, is high. Lately, rotavirus has also been detected more frequently in adults [14,15]. Perhaps, testing for rotavirus for adult patients can be considered if no other pathogens have been detected. Also, since rotavirus can cause nosocomial spread following hospital admittance, diagnosing rotavirus could be important for public health reasons. No attention is given in the guidelines to the risk of spreading a viral infection.

Compared with a previous physician-based survey [6], virus tests were underrepresented in the current study. This might be because routine laboratories are not (yet) able to test for norovirus, which frequently is the cause of acute gastroenteritis [6]. Also rotavirus and adenovirus were not often requested. Routinely testing for some viruses yielded a 5% positivity rate for rotavirus and 2% for adenovirus [6], which was only 0.8 and 0.4% respectively in the current study. The reason for this difference, which was not observed for the other pathogens, might be that the GPs requested tests for viruses most often in the summer months, while rotavirus is a frequent cause of gastroenteritis in the winter months.

In short, the GPs only partially followed the guidelines. More adherence to these guidelines should be promoted. As these guidelines were set up in 1996, they need to be updated with the results of studies conducted since then, which especially demonstrated the importance of viral infections such as rotavirus and norovirus. This study has shown that GPs are not always aware of the importance of viral causes of acute gastroenteritis, according to the stool tests they requested. Rotavirus plays an important role for children aged 0 to 5 years, especially in the winter months. A test for Shigella should only be requested for patients with a travel history. Subsequently, it will be necessary to direct the GPs more into implementing the revised guidelines.

## Conclusion

In conclusion, about 1 in 8 patients consulting their GP for gastroenteritis will be requested to submit a stool sample for laboratory testing. Practices for requesting stools by GPs were sometimes contradictory with results from previous studies. For example, viruses were relatively seldom requested, and if so, preferentially after the seasonal peak and in lower risk age groups. Also the guideline to request protozoa tests for travel-related and longterm cases is not consistently followed by the GPs. Thus, data of this study can be used to develop educational approaches for GPs to improve their practices for gastroenteritis patients. Beforehand, the sometimes outdated guidelines provided to the GPs need to be revised (table 6).

**Table 6:** Conclusions

**What is known?**	**What is new?**
• Gastroenteritis is a common disease in the Netherlands	• In the Netherlands, for patients with acute gastroenteritis, relatively few stool samples are requested by GPs when compared to the United States, Canada and the United Kingdom
• Viruses are the most important causes of gastroenteritis	• The reason for a request for a stool sample are related to age: 0 to 4 years: reassurance of parents; 40 to 64 years: recent visit to a specific country; 65 years and older: duration or severity of complaints
• In the Netherlands, patients with acute gastroenteritis visit their GP less often when compared to patients in the United Kingdom	• Test requests did not always comply with existing knowledge of the etiology of gastroenteritis
	• The Dutch National guidelines for acute gastroenteritis should be revised

## Competing interests

The authors declare that they have no competing interests.

## Authors' contributions

WB en YD performed the data analysis. YD also developed the study concept. AIMB headed the study coordination. MK contributed to the manuscript.

All authors read and approved the final manuscript.

## Funding

The Dutch ministry of Public Health, Welfare and Sports funded the survey.

## Pre-publication history

The pre-publication history for this paper can be accessed here:


